# O’Leary-Sant Symptom Index Predicts the Treatment Outcome for Onabotulinumtoxin A Injections for Refractory Interstitial Cystitis/Bladder Pain Syndrome

**DOI:** 10.3390/toxins7082860

**Published:** 2015-07-30

**Authors:** Yuh-Chen Kuo, Hann-Chorng Kuo

**Affiliations:** 1 Department of Urology, Yangming Branch of Taipei City Hospital, 105 Yu-Sheng Street, Taipei 11148, Taiwan; E-Mail: yuhchens@hotmail.com; 2 Department of Urology, Buddhist Tzu Chi General Hospital and Tzu Chi University, 707, Section 3, Chung Yang Road, Hualien 97002, Taiwan

**Keywords:** interstitial cystitis, onabotulinumtoxin A, treatment, predictive factors

## Abstract

Although intravesical injection of onabotulinumtoxinA (BoNT-A) has been proved promising in treating patients with interstitial cystitis/bladder pain syndrome (IC/BPS), what kind of patients that may benefit from this treatment remains unclear. This study investigated the predictors for a successful treatment outcome. Patients with IC/BPS who failed conventional treatments were enrolled to receive intravesical injection of 100 U of BoNT-A immediately followed by hydrodistention. Variables such as O’Leary-Sant symptom and problem indexes (ICSI and ICPI), pain visual analogue scale (VAS), functional bladder capacity (FBC), voiding diary, and urodynamic parameters were measured at baseline and six months after treatment. A global response assessment (GRA) ≥ 2 at six months was defined as successful. There were101 patients enrolled. Significant improvements were observed in mean ICSI, ICPI, OSS (ICSI + ICPI), pain VAS, FBC, frequency, nocturia and GRA at six months after BoNT-A injections (all *p* < 0.05). The successful rate at six months was 46/101 (45.54%). Multivariate logistic regression revealed the baseline ICSI (odds ratio = 0.770, 95% confidence interval = 0.601–0.989) was the only predictor for a treatment outcome. ICSI ≥ 12 was the most predictive cutoff value for a treatment failure, with a ROC area of 0.70 (sensitivity = 69.1%, specificity = 60.9%).

## 1. Introduction

Interstitial cystitis/bladder pain syndrome (IC/BPS) is a debilitating chronic disease of unknown etiology characterized by urgency, frequency, and suprapubic discomfort during bladder filling. Current treatments are usually unsuccessful in completely eradicating bladder pain and increasing bladder capacity [1]. Intravesical resiniferatoxin once was considered to be effective but this has not been shown in a large scale multiple center trial [2]. Other intravesical therapies such as hyaluronic acid, bacilli Calmette-Guerine, and oral medications with pentosan polysulfate sodium, cyclosporine A, or amitriptyline have not been demonstrated to be long-term effective [3,4,5,6]. Until now, bladder hydrodistention is still the most popular treatment for IC/BPS refractory to conventional treatment. However, the effective duration of hydrodistention is usually short and repeated hydrodistention or conversion to other treatment such as botulinum toxin injection is necessary.

Onabotulinumtoxin A (BoNT-A) is one of the most powerful neurotoxins to inhibit the release of neurotransmitters from nerve fibers and urothelium [7,8]. Applications of BoNT-A for IC/BPS have been reported in some studies [9,10,11,12,13]. The results showed BoNT-A could reduce bladder pain, impair bladder sensation, and decrease chronic inflammation in the central nervous system in animal and human experiments. Although BoNT-A injection seems promising for treating symptoms of IC/BPS [14], most of the results are based on the data of mean changes of symptoms collected from the entire study cohort. There is still a certain percentage of patients who had unsuccessful outcomes after treatment. So far, which kind of patients will benefit from BoNT-A injection remains unclear.

Identification of the predictive factors for a successful treatment outcome may help differentiating the subset of IC/BPS patients who will not respond to single BoNT-A injection. We could then avoid giving these patients ineffective procedures and exposing them to potential treatment-related adverse effects. Additionally, we might further counsel and encourage them to receive more advanced treatment options such as repeated BoNT-A injections or enterocystoplasty. We conducted this study to evaluate if there are clinical variables that can predict a treatment success after a single injection of BoNT-A for the treatment of IC/BPS refractory to conventional treatment.

## 2. Results and Discussion

From October 2005 to October 2013, 101 patients with diagnosis of IC/BPS who have failed conventional treatments were enrolled in this study. Among them, there were 13 men and 88 women who aged 46.00 ± 13.27 (27–72) and 48.81 ± 11.81 (21–78) years, respectively. The demographics of these subjects are described in Table 1. There was no significant difference between either sex in distribution of age, IC symptom scores, pain visual analogue scale (VAS), functional bladder capacity (FBC), daytime frequency, nocturia, urodynamic parameters, result of potassium sensitivity test (PST) and cystoscopic parameters.

**Table 1:** Demographics of the patients.

Parameters	Total (*n* = 101)	Male (*n* = 13)	Female (*n* = 88)	*p* Value
Age (years)	48.45 ± 11.97	46.00 ± 13.27	48.81 ± 11.81	0.433
ICSI	12.34 ± 3.42	11.08 ± 3.95	12.52 ± 3.32	0.156
ICPI	11.43 ± 3.03	11.77 ± 3.19	11.38 ± 3.02	0.664
OSS (ICSI + ICPI)	23.76 ± 6.14	22.85 ± 6.59	23.90 ± 6.09	0.567
Pain VAS	5.23 ± 2.37	4.23 ± 3.24	5.38 ± 2.20	0.105
FBC	128.86 ± 75.37	156.92 ± 58.93	124.72 ± 76.91	0.151
Frequency	15.39 ± 7.68	13.69 ± 6.10	15.64 ± 7.88	0.397
Nocturia	4.70 ± 4.67	3.38 ± 1.85	4.90 ± 4.93	0.278
FSF	115.96 ± 53.40	105.92 ± 55.63	117.33 ± 53.28	0.490
FD	171.19 ± 73.44	192.20 ± 72.48	168.53 ± 73.59	0.340
SD	202.46 ± 87.01	233.67 ± 79.81	197.45 ± 87.74	0.249
CBC	273.98 ± 109.32	268.83 ± 109.71	274.72 ± 109.92	0.863
Pdet	19.87 ± 10.82	24.33 ± 11.39	19.19 ± 10.65	0.126
Qmax	12.56 ± 5.38	12.17 ± 4.86	12.62 ± 5.48	0.787
Volume	241.18 ± 110.54	219.67 ± 101.13	244.41 ± 112.12	0.473
PVR	39.38 ± 94.08	49.17 ± 103.61	38.01 ± 93.26	0.703
Positive PST	98 (97.03)	12 (92.31)	86 (97.73)	0.342 ^1^
Cystoscopic HD				
MBC (mL)	570.00 ± 198.07	603.85 ± 145.00	681.82 ± 218.30	0.216
Hunner’s ulcer	10 (9.90)	0 (0)	10 (11.36)	0.200 ^1^
Glomerulation				0.386 ^1^
Grade 0	8 (7.92)	1 (7.69)	7 (7.95)	
Grade 1	39 (38.61)	7 (53.85)	32(36.36)	
Grade 2	35 (34.65)	3 (23.08)	32 (36.36)	
Grade 3	11 (10.89)	0 (0)	11 (12.50)	
Grade 4	8(7.92)	2 (15.38)	6 (6.82)	

Note: ^1^ Chi-square test. Otherwise, the independent *t* test was used; ICSI: O’Leary-Sant IC Symptom Index; ICPI: IC Problem Index; VAS: Visual analogue scale. FBC: Functional bladder capacity; FSF: First sensation of filling; FD: First desire to void. SD: Strong desire to void; CBC: Cystometric bladder capacity; Pdet: Detrusor pressure at Qmax; Qmax: Maximum flow rate; PVR: Postvoid residual; PST: Potassium sensitivity test; HD: Hydrodistention. MBC: Maximal bladder capacity.

Table 2 listed the changes of clinical parameters at baseline, three and six months after a single injection of BoNT-A. For all of the patients, significant improvements could be observed in mean O’Leary-Sant IC Symptom Index (ICSI) and IC Problem Index (ICPI), OSS (ICSI + ICPI), pain VAS, FBC, frequency, nocturia, and global response assessment (GRA) at three months after BoNT-A injections. These improvements continued to exist at six months which demonstrated the durable efficacy of BoNT-A injection on the treatment of refractory IC/BPS.

**Figure 1:**
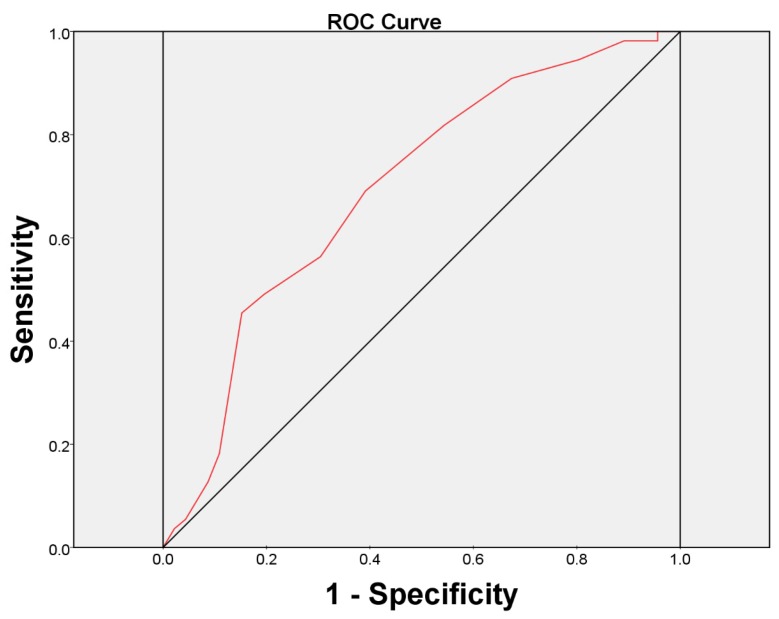
The area under the receiver operating characteristic (ROC) curve for baseline ICSI score ≥ 12 as a prognostic test for the patients with a failed treatment outcome.

At six months after BoNT-A injection, the overall therapeutic results were reported to be failed in 55 (54.46%) and successful in 46 (45.54%) patients by GRA criteria (GRA ≥ 2 as successful) (Table 3). Univariate logistic regression analysis revealed significant relationships between the treatment outcome and baseline ICSI, ICPI, OSS, FBC, frequency, and the urodynamic parameter, first desire to void (FD) (all *p* < 0.05). That is, the subjects with lower ICSI, ICPI, OSS scores, lower frequency times, higher mean amount of FBC or higher mean volume of FD at baseline had a significantly greater chance to achieve a successful treatment outcome. In addition, the multivariate logistic regression model was performed based on all significant variables in the univariate analysis and showed that the baseline ICSI score (odds ratio = 0.770, 95% confidence interval = 0.601–0.989) was the only predictive factor for a treatment outcome in refractory IC/BPS patients (Table 4). We found ICSI ≥ 12 was the most predictive cutoff value for treatment failure, with a ROC area of 0.70 (sensitivity = 69.1%, specificity = 60.9%) (Figure 1).

**Table 2:** The changes of Parameters at baseline, 3 and 6 months after single BoNT-A injection.

Parameters (*N* = 101)	Baseline	3 Months	6 Months	*p* Value ^1^
ICSI	12.34 ± 3.42	8.92 ± 4.42	8.79 ± 4.65	0.000
ICPI	11.43 ± 3.03	7.51 ± 4.30	7.75 ± 4.57	0.000
OSS (ICSI+ICPI)	23.76 ± 6.14	16.43 ± 8.51	16.54 ± 8.95	0.000
Pain VAS	5.23 ± 2.37	3.45 ± 2.47	3.50 ± 2.54	0.000
FBC	128.86 ± 75.37	180.00 ± 90.53	177.92 ± 86.39	0.000
Frequency	15.39 ± 7.68	11.79 ± 6.35	11.28 ± 6.29	0.000
Nocturia	4.70 ± 4.67	3.31 ± 2.86	3.52 ± 3.79	0.026
Qmax	12.56 ± 5.38	13.97 ± 6.07	14.53 ± 5.91	0.060
Volume	241.18 ± 110.54	236.66 ± 123.27	236.50 ± 121.70	0.955
PVR	39.38 ± 94.08	48.03 ± 79.09	48.09 ± 77.48	0.711
CBC	273.98 ± 109.32	285.64 ± 140.59	284.59 ± 142.12	0.799
GRA	0	1.27 ± 1.00	1.32 ± 0.97	0.000

Note: ^1^ Wilcoxon rank-sum test; ICSI: O’Leary-Sant IC Symptom Index; ICPI: IC Problem Index; VAS: Visual analogue scale; FBC: Functional bladder capacity; Qmax: Maximum flow rate; PVR: Postvoid residual; CBC: Cystometric bladder capacity; GRA: Global response assessment.

**Table 3:** Univariate logistic regression of parameters associated with the treatment outcome of intravesical BoNT-A injection for IC/BPS.

Parameters	Treatment Outcome		OR (95% CI)	*p* Value
	Successful	Failed		
No. patients	46	55		
Gender				
Male (%)	5 (38.46)	8 (61.54)	1.396 (0.423–4.603)	0.584
Female (%)	41 (46.59)	47 (53.41)		
Age (years)	46.91 ± 12.00	49.73 ± 11.91	0.980 (0.948–1.014)	0.240
ICSI	11.15 ± 3.47	13.33 ± 3.07	0.811 (0.709–0.928)	0.002
ICPI	10.76 ± 3.34	11.98 ± 2.65	0.869 (0.756–0.999)	0.048
OSS (ICSI+ICPI)	21.91 ± 6.56	25.31 ± 5.35	0.905 (0.756–0.999)	0.008
Pain VAS	4.80 ± 2.25	5.58 ± 2.43	0.866 (0.729–1.030)	0.104
FBC	151.96 ± 82.75	109.55 ± 63.09	1.008 (1.002–1.014)	0.007
Frequency	13.37 ± 5.02	17.07 ± 9.04	0.922 (0.861–0.989)	0.023
Nocturia	3.59 ± 2.54	5.64 ± 5.75	0.852 (0.724–1.002)	0.052
VUDS parameters				
FSF (mL)	123.93 ± 49.57	109.17 ± 56.03	1.005 (0.998–1.013)	0.169
FD (mL)	191.12 ± 68.71	154.17 ± 73.74	1.007 (1.001–1.014)	0.021
SD (mL)	222.64 ± 83.10	181.66 ± 87.31	1.006 (1.000–1.012)	0.062
CBC (mL)	289.50 ± 107.99	259.41 ± 109.66	1.003 (0.999–1.006)	0.181
Pdet (cm H_2_O)	19.51 ± 10.39	20.19 ± 11.30	0.994 (0.957–1.033)	0.765
Qmax (mL/s)	13.03 ± 4.81	12.11 ± 5.89	1.033 (0.955–1.117)	0.416
Voided volume (mL)	264.82 ± 110.28	219.52 ± 107.38	1.004 (1.000–1.008)	0.052
PVR (mL)	32.60 ± 91.79	42.19 ± 96.87	0.999 (0.995–1.004)	0.752
PST				
Negative (%)	1 (33.33)	2 (66.67)		
Positive (%)	45 (45.92)	53 (54.08)	1.698 (0.149–19.349)	0.670
Cystoscopic HD				
MBC (mL)	688.04 ± 199.77	658.18 ± 221.48	1.001 (0.999–1.003)	0.478
Hunner’s ulcer				
Negative (%)	43 (47.25)	48 (52.75)		
Positive (%)	3 (30.00)	7 (70.00)	0.307 (0.756–0.999)	0.307
Glomerulation				
Grade 0 (%)	6 (75.00)	2 (25.00)		
Grade 1 (%)	15 (38.46)	24 (61.54)	0.208 (0.037–1.170)	0.075
Grade 2 (%)	18 (51.43)	17 (48.57)	0.353 (0.062–1.995)	0.239
Grade 3 (%)	4 (36.36)	7 (63.64)	0.190 (0.025–1.432)	0.107
Grade 4 (%)	3 (37.50)	5 (62.50)	0.200 (0.023–1.712)	0.142

Note: OR: Odds ratio; CI: Confidence interval; ICSI: O’Leary-Sant IC Symptom Index; ICPI: IC Problem Index; VAS: Visual analogue scale; FBC: Functional bladder capacity; FSF: First sensation of filling; FD: First desire to void; SD: Strong desire to void; CBC: Cystometric bladder capacity; Pdet: Detrusor pressure at Qmax; Qmax: Maximum flow rate; PVR: Postvoid residual; PST: Potassium sensitivity test; HD: Hydrodistention; MBC: Maximal bladder capacity.

**Table 4:** Multivariate analysis of parameters significantly associated with a successful treatment outcome on univariate analysis.

Parameters	Odds Ratio	95% CI	*p* Value
ICSI	0.770	0.601–0.989	0.040
ICPI	1.106	0.853–1.435	0.447
FBC (mL)	1.003	0.996–1.010	0.389
Frequency	1.011	0.904–1.053	0.531
FD (mL)	1.005	0.998–1.012	0.171

Note: CI: Confidence interval; ICSI: O’Leary-Sant IC Symptom Index; ICPI: IC Problem Index; FBC: Functional bladder capacity; FD: First desire to void.

So far the pathogenesis of IC/BPS is not fully understood. The management of IC/BPS is directed mainly to amelioration of the bothersome symptoms such as bladder pain or urinary tract symptoms. This study demonstrated that intravesical BoNT-A injection could significantly relieve the bothersome symptoms (ICSI, ICPI, OSS, pain VAS, daytime frequency and nocturia) and increase the functional bladder capacity in patients with refractory IC/BPS at 3 and 6 months after treatment. These effects are comparable to previous reports [11,12,13,15].

BoNT-A acts by cleaving the SNAP-25 (Synaptosome-associated protein of 25 kd) complex in the presynaptic terminal, which prevents formation of the SNARE system. By this mechanism, the neurotransmitter vesicles cannot work at the presynaptic membrane, which decreases the release of neurotransmitters at the synaptic cleft. Consequently, the release of acetylcholine, calcitonin gene-related protein, substance-P, and glutamate decreased and the nociceptive fiber discharge reduced [16,17], suggesting the therapeutic rationale of BoNT-A on IC/BPS. Furthermore, recent studies have demonstrated that BoNT-A has an anti-inflammatory effect on a cystitis rat model [18] and that injection of BoNT-A can reduce the production of nerve growth factor in bladder resulting in satisfactory pain relief in IC/PBS patients [19,20].

Pilot studies of intravesical injection of BoNT-A for IC/BPS had been introduced since 2004 with variable successful rates. Smith *et al.* reported a 69% success rate with a therapeutic duration of nine months [9]. Giannantoni *et al.* reported 85.7% of patients had an improvement at three months [11]. Our previous study revealed that single intravesical injections of BoNT-A, followed by hydrodistention, produced significantly better clinical results than hydrodistention alone [21]. Of the patients 71% had a successful result at six months. In the current study, the overall success rate of treatment at six months was 45.54% according to GRA ≥ 2. The large variation in successful rates between the above studies may reflect the differences of the study population, disease severity, definition of success, dosage of BoNT-A, injection method, and so on, in different study cohorts. However, these reports are similar in one fact: not all the IC/BPS patients benefit from BoNT-A injection. This result implies the need for further research on the causes of a treatment failure or the ways to differentiate which IC/BPS patient will take the advantage of BoNT-A injection.

Based on our findings from univariate logistic regression analysis, the patients who failed BoNT-A injection at six months possessed higher baseline ICSI, ICPI, OSS, frequency, and lower mean volume of FBC and FD than those who succeeded in treatment. However, using multivariate logistic regression model, baseline ICSI was found to be the only independent predictive factor for a successful treatment of BoNT-A injection. The reason for this is not clear. It is possible that a higher symptom score (ICSI ≥ 12) may reflect more advanced urothelial dysfunction and neurogenic inflammation of the bladder leading to more unpleasant sensation during bladder filling, and possibly more severe fibrosis of the bladder wall which could not be resolved completely by single injection of BoNT-A. Since the components of ICSI questionnaire consist of four domains, urgency, frequency, nocturia, and pain, our finding suggested that the overall patients’ perception, ICSI, but not individual symptom or clinical variable before treatment will predict the treatment outcome of BoNT-A injection for refractory IC/BPS. To our knowledge, this is the first study to discover the prognostic factor of intravesical BoNT-A injection for the treatment of IC/BPS.

The results of this study also point out that only 45.54% of patients have a successful treatment from a single BoNT-A injection. Although BoNT-A has anti-inflammation and anti-nociceptive effects, a single injection might not completely resolve the chronic inflammation residing in the bladder wall. This result is also reflected by the previous studies that the therapeutic effect is fading with time and no patient can have effect at 12 months after 200 U BoNT-A single injection [12], and repeated BoNT-A injections provides a significantly better long-term effect compared with a single injection [22,23].

Recently, Denys *et al.* observed that neurogenic detrusor overactivity (NDO) patients without response at a first onabotulinumtoxin injection had a good response at a second injection [24]. Also, NDO patients who failed after abobotulinum toxin in 20 sites had good responses after an equivalent onabotulinum toxin injection in 30 sites [25]. The quality of injections and reconstitution of onabotulinum toxin may have an influence on the effect of the first BoNT-A injection. As such, one may argue that we can assume failure after a single injection. In our study, all the injections were prepared and performed by a single operator (Kuo, H.C.) with more than ten years’ experience in BoNT-A injection. The treatment procedure has been standardized and risk of inappropriate injection has been minimized so that the treatment outcome can be compared on this basis.

During bladder inflammation, the exocytosis of TRPV1 increases and plays a role in the perception of thermal and inflammatory pain [26]. BoNT-A could block the TRPV1 trafficking to the membrane during bladder inflammation and inhibit the inflammatory sensitization of TRPV1 [27]. Intravesical onabotulinumtoxin A administration could inhibit the evoked ATP release from urothelium of chronic bladder inflammation models [28]. BoNT-A administration to the rat bladder can also decrease the amount of spinal cord C-fos expression due to chronic bladder inflammation [29]. One proposed mechanism of BoNT-A induced analgesia is the combination of direct analgesic effect by its action on peripheral nociceptive neurons and indirect analgesic affect by the retrograde transport of toxin to prevent central sensitization in patients with chronic pain [30]. A recent randomized, double-blind, placebo-controlled, multicentre trial showed that bladder injections of 100 U of BoNT-A effectively reduced bladder pain symptoms in patients with IC/BPS. At week eight, a significantly greater reduction in pain score was observed in the BoNT-A group compared to the normal-saline group (−2.6 ± 2.8 *vs.* −0.9 ± 2.2, *p* = 0.021). Adverse events did not differ between the groups [31].

The major limitations of our present study included the lack of a control arm and that it was not randomized. Although an ICSI score of 12 was found to be the best cutoff value for predicting therapeutic efficacy, the clinical implication of this cutoff value is limited by its small area under the ROC curve, and its low sensitivity and specificity. We should realize that even in patients with high ICSI, some may have a good treatment response. Nevertheless, these findings could serve as an initial guide or assist in consultation regarding the treatment of IC/BPS patients with BoNT-A. Further well-designed randomized trials to confirm the results of this study are needed.

## 3. Experimental Section

### 3.1. Patient Enrollment

Patients with IC/BPS who have failed conventional treatments have been enrolled in this study. They have been treated with oral pentosan polysulfate sodium, intravesical instillation of heparin, hyaluronic acid, or oral tricyclic antidepressant for at least six months, but the symptoms remained unchanged or relapsed. A diagnosis of IC/BPS has been established based on characteristic symptoms and cystoscopic findings of glomerulation, petechia, or mucosal ulceration [32].

### 3.2. Outcome Assessment

Patients were requested to complete a three-day voiding diary before and after treatment. The IC/BPS symptoms were assessed by the O’Leary-Sant IC Symptom Index (ICSI) and IC Problem Index (ICPI) [33]. The pain score was reported by patient self-assessment using a 10-point visual analogue scale (VAS) system. Video-urodynamic study (Video UDS) and PST were performed. The treatment outcome was assessed using the GRA [34]. Patients were requested to rate symptoms compared with baseline on a seven-point centered scale from markedly, moderately and slightly worse, no change, to slightly, moderately and markedly improved. Patients with moderately and markedly improved results after treatment (GRA ≥ 2) were considered to have a successful treatment outcome. Otherwise, the treatment was considered to have failed.

### 3.3. Urodynamic Study

UDS was performed by standard procedures using a 6 Fr dual channel catheter and an 8 Fr rectal balloon catheter. Cystometric study was performed with normal saline at a filling rate of 20 mL/min. All descriptions and terminology in this report were in accordance with the recommendations of the International Continence Society [35]. The urodynamic parameters included first sensation of bladder filling (FSF), first desire to void (FD), strong desire to void (SD), cystometric bladder capacity (CBC), maximum flow rate (Qmax), detrusor pressure at Qmax (Pdet) and postvoid residual (PVR). After the VUDS, 40 mL KCl solution of 0.4 M was infused slowly into the bladder and the test was regarded as positive when painful (of ≥2 VAS score) or urgency sensation was elicited compared to normal saline infusion during urodynamic study.

### 3.4. Botulinum Toxin Injections

The patients were admitted for the treatment. They received intravesical injection of 100 U of BoNT-A (BOTOX, Allergan, Irvine, CA, USA) immediately followed by cystoscopic hydrodistention under intravenous general anesthesia. Each vial of BoNT-A was diluted with 10 ml of normal saline, resulting in 10 U BoNT-A per 1.0 mL. Patients received 20 sites of suburothelial injection, which contained 5 U in 0.5 mL for each site. The injection needle was inserted about 1 mm into the urothelium at the posterior and lateral walls of the bladder, using a 23 gauge needle and rigid cystoscopic injection instrument (22 Fr, Richard Wolf, Knittlingen, Germany). Cystoscopic hydrodistention was performed to an intravesical pressure of 80 cm water for 15 min immediately after the injection and the maximal bladder capacity (MBC) under hydrodistention was recorded.

### 3.5. Patient Follow-up

The patients were followed up every three months after treatment. The score of ICSI and ICPI, information on functional bladder capacity (FBC), daily urinary frequency, nocturia, pain VAS, and UDS parameters were recorded. At six months after the initial BoNT-A injection the patients were questioned of bladder conditions.

### 3.6. Statistical Analysis

The results of the voiding diary, UDS, ICSI, ICPI, and pain VAS were compared between baseline and each follow-up time point. Statistical comparisons between the groups were tested using a chi-square test for categorical variables, and an independent *t* test or a Wilcoxon rank-sum test for continuous variables. Univariate and multivariate logistic regression analyses were used to identify variables predicting treatment success at six months after BoNT-A injection. The most predictive cutoff value of each variable was determined by the receiver operating characteristic curve (ROC) analysis. Statistical assessments were considered significant when *p* < 0.05. Statistical analyses were performed using SPSS 18.0 statistical software (SPSS Inc., Chicago, IL, USA).

## 4. Conclusions

Baseline ICSI score represents the independent predictor for treatment success after BoNT-A injection. Patients with an ICSI of 12 or greater may have a poorer treatment outcome.
